# Nematophagous fungi as biological control agents of parasitic nematodes in soils of wildlife parks

**DOI:** 10.1016/j.ijppaw.2024.101033

**Published:** 2024-12-12

**Authors:** Christopher Sander, Stephan Neumann

**Affiliations:** Institute of Veterinary Medicine, Georg-August-University of Goettingen, Burckhardtweg 2, 37077, Goettingen, Germany

**Keywords:** Soil-transmitted helminths, Nematodes, Predatory fungi, Wildlife, Pest management

## Abstract

Infections with soil-transmitted helminths pose a significant threat to wildlife in enclosures, where transmission of these parasitic larvae is easier due to the limited space. Nematophagous fungi offer a promising solution as they can naturally control these nematodes. In this study, three nematophagous fungi (*Arthrobotrys oligospora*, *Dactylaria scaphoides*, *Nematoctonus leiosporus*) purchased from the non-profit global biological resource center ATCC were tested for their suitability as biological control agents. The nematodes *Strongyloides* sp., *Trichostrongylus* sp. and *Oesophagostomum* sp. Were isolated from three animal species: wild boar (*Sus scrofa*) (n = 10), fallow deer (*Dama*) (n = 5) and mouflon (*Ovis orientalis musimon*) (n = 5) from a wildlife park using the Baermann-Wetzel method. In a second step, the fungi were brought into contact with the parasites on the soil of the enclosures. The two media tests showed that the nematophagous fungi were more effective on the agar plate than on the autoclaved soil. Only *D. scaphoides* showed good efficacy on both media, while the other two fungi showed more marked differences on the two media. The results showed that these three nematophagous fungi can reduce parasites in soil before they are ingested by an infected animal. Given the increasing development of drug resistance and the use of chemical agents for soil treatment, this is an important finding that should be pursued in the future.

## Highlights

• Investigation of three nematophagous fungi as biological control agents
• Focus on control of parasitic larvae already in the soil instead of in the animal
• Tested on two different media: agar plates and enclosure floors
• Interaction with three nematode species investigated
• *Dactylaria scaphoides* has good potential as a biological control agent

## Graphical abstract

**Figure:**
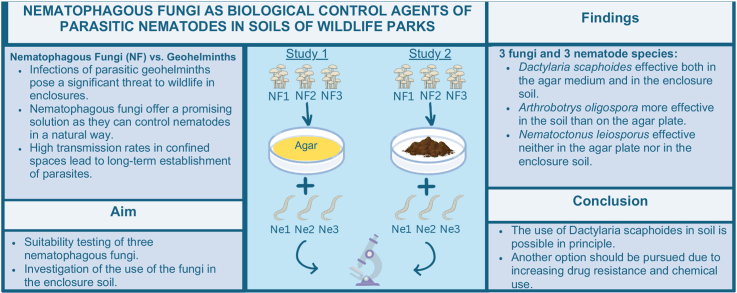

## 1 Introduction

The ecological health of soils in wildlife parks is critical to maintaining the biodiversity that lives in and on them. However, soil-transmitted helminths (STH), worm-like parasites, pose a significant threat to wildlife in enclosures. These parasites, which are widely distributed and capable of causing severe infections, colonize the gastrointestinal tract, liver, and other organs of their hosts. Infected animals can suffer from weight loss, reduced reproductive capacity, and even death. The transmission cycle begins when parasitic worms release eggs in the feces of infected hosts. These eggs contaminate the soil, where they mature, and are then ingested by other animals, leading to new infections. Once inside the host, adult worms reside in the intestines, producing thousands of eggs daily (Coulson et al., 2018; Esteban-Sánchez et al., 2024; Panayotova-Pencheva, 2013).

Wild animals in enclosures play a central role in the life cycle of these parasites, as they serve as hosts for the development and spread of the nematodes. The constant cycle of infection and reinfection leads to a high parasite load in the soil and in the animals, which requires effective and sustainable control (Barbosa et al., 2020; Palomero et al., 2020).

Conventional chemical control of parasitic nematodes, such as the use of quicklime, often has negative effects on the environment and non-parasitic soil organisms (Capizzi-Banas et al., 2004). In addition, the regular use of anthelmintics can lead to the development of resistance in the parasites. This development of resistance poses an increasing challenge as the efficacy of anthelmintics decreases and higher doses or alternative drugs are required, which in turn can cause additional environmental problems. This emphasises the need for environmentally friendly alternatives (Bellaw and Nielsen, 2015; Eysker and Ploeger, 2000; Palomero et al., 2020; Saumell et al., 2015).

A promising ecological solution is offered by nematophagous fungi that can naturally control parasitic nematodes. These fungi have special mechanisms, such as traps and toxins, to infect and kill nematodes. Studies on nematophagous fungi focus both on the use of the fungi in animals and on contaminated soils (Li et al., 2022; Longo Ribeiro Vilela et al., 2016; Luns et al., 2018; Palomero et al., 2020; Szewc et al., 2021).

In our opinion, the focus on the enclosure soils appears to be more decisive, as control there can better reduce the likelihood of infection by reducing parasites in the soil.

Therefore, fecal samples were collected from infected animals in a wildlife park and the parasite larvae were isolated using the Baermann-Wetzel method. The aim of this study was to test a new approach to the treatment of enclosure soils. Therefore, the suitability and efficiency of the three nematophagous fungi *Arthrobotrys oligospora*, *Dactylaria scaphoides* and *Nematoctonus leiosporus* as sustainable and ecological control of soil-borne helminths in wildlife was investigated. For this purpose, a comparative two-stage study on the interaction between fungi and parasites was conducted: first on culture media (agar) and then on enclosure floors.

By promoting and utilising these natural enemies of nematodes, sustainable nematode control strategies can be developed that both improve wildlife health and protect the environment.

## 2 Material and methods

### 2.1 Fungi and production of mycelial mass

To investigate the suitability of nematophagous fungi as biological control agents, three nematophagous fungi were obtained from the non-profit, global biological resource center ATCC (American Type Culture Collection): *Arthrobotrys oligospora* (ATCC 24927), *Dactylaria scaphoides* (ATCC 38780), and *Nematoctonus leiosporus* (ATCC 36923). These fungi are known nematode trappers.

The inoculum containing viable cells of *D. scaphoides* was spread on Potato Carrot Agar, half-strength. For *A. oligospora* and *N. leiosporus*, the inocula containing viable cells were spread on Potato Dextrose Agar (PDA). The inoculated media were then incubated at a constant temperature of 25 °C for 28 days to allow mycelial growth. Growth was monitored regularly to ensure the development of a robust mycelial mass.

### 2.2 Isolation of soil-transmitted helminths

Twenty fecal samples were collected in 2023 from a wildlife park in southern Lower Saxony. The fecal samples were obtained from three animal species: wild boar (*Sus scrofa*) (n = 10), fallow deer (*Dama*) (n = 5), and mouflons (*Ovis orientalis musimon*) (n = 5). These animals were known to be infected with soil-transmitted helminths (STHs).

To prepare the isolation of nematodes L3, a larval culture was carried out for 10 days, similar to (Dashe and Berhanu, 2020). For this purpose, about 10 g of feces were mixed with tap water and crushed wood pellets in a mortar to form a homogeneous suspension and left to rest in a Petri dish for 10 days. After this time, the L3 larvae were extracted using the Baermann apparatus. For this purpose, part of the suspension was passed through a sieve into a funnel and left to rest for 2 h. Then about 15 ml was poured into a 50 ml vessel. After standing for 1 h, the water was decanted and refilled. After a further hour, the water was decanted again. Then about 120 μL was removed from the sediment with a pipette and placed on a slide with a well (15–18 mm diameter, 0.6–0.8 mm depth) to be examined under the microscope. The coverslip was systematically scanned at low magnification (10–20x) in meandering paths from left to right and magnified at high magnification (40–60x) when signs of nematode L3 were detected. The larvae were then removed from the slide using a 20 μL pipette and collected in an Eppendorf tube. The isolation of the nematodes merely served as preparation for the investigation of the interactions between the nematodes and the nematophagous fungi. After examining the fecal samples, the three most common parasites of the observed wild animals were selected: *Strongyloides* sp., *Trichostrongylus* sp. and *Oesophagostomum* sp.

The identification of the larvae were done using the book Koprologische Diagnostik von Endoparasiten in der Veterinärmedizin by Schmäschke (2014), article by Van Wyk and Mayhew (2013) and the identification charts and literature of the laboratory (Schmäschke, 2014; Van Wyk and Mayhew, 2013).

### 2.3 Test design

A two-stage test design was developed to investigate the efficiency of three nematophagous fungal strains in capturing and killing three parasitic nematode larvae species. The first series of tests was used to evaluate the suitability of the fungi on agar plates, while the second series of tests performed the same investigation on autoclaved enclosure soils. Both approaches were chosen to analyze the interactions under controlled laboratory conditions and under simulated environmental conditions.

Three repetitions and one control (nematodes without fungi) were carried out in each row. For each fungus, 50 nematodes L3 of the three most frequently isolated species were added to the medium.

#### 2.3.1 Suitability of the fungi on agar plates

**Fig. 1:**
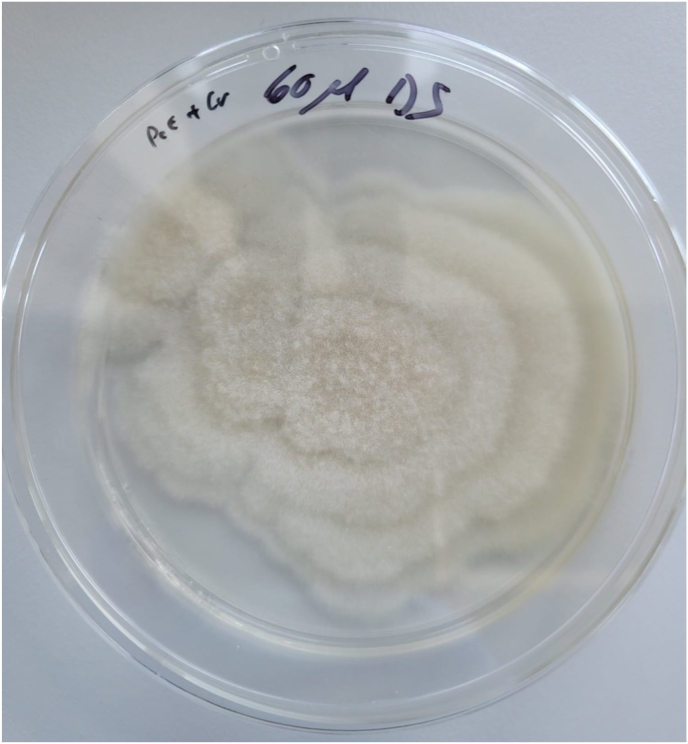
Growth of *Dactylaria scaphoides* on potato-carrot agar (half-strength) medium.

In preparation for the series of experiments with the agar plates, sterile Petri dishes were poured with Potato Carrot Agar (half-strength) and Potato Dextrose Agar (PDA). After hardening, the plates were inoculated with the already cultivated nematophagous fungal strains (*A. oligospora*, *D. scaphoides*, *N. leiosporus*) according to their cultivation media. A standardised amount of fungal mycelium (approx. 5 g) was used as inoculum for this purpose. The fungal mycelium was placed in the center of the agar plates and the new plates were then incubated at 25 °C for 7 days to ensure extensive mycelial growth (Fig. 1). After incubation, 50 larvae L3 of each of the three isolated nematode species (*Strongyloides* sp., *Trichostrongylus* sp., and *Oesophagostomum* sp.) were placed on the agar plates near the fungal mycelium. The plates were examined microscopically every two days, and the number of captured and killed nematodes after 14 days was documented. Each combination of fungus and nematode was replicated three times to ensure statistical significance of the results. The efficiency of the fungi was calculated by the number of captured and killed nematodes and statistically analyzed.

#### 2.3.2 Suitability of fungi on enclosure soils

For the second series of experiments, enclosure soils were sterilized by autoclaving at 121 °C for 45 min. Subsequently, 30 g of soil was evenly distributed per sterile Petri dish. The soils were then inoculated with the three fungal strains (*A. oligospora*, *D. scaphoides*, *N. leiosporus*) by placing approx. 5 g of fungal mycelium in the center of the sterile soil. The fungi on the soil were then incubated for another 7 days. After the 7 days, 50 larvae L3 of the three isolated nematode species (*Strongyloides* sp., *Trichostrongylus* sp., and *Oesophagostomum* sp.) were added to each enclosure soil dish. Soil samples were taken after 14 days and the nematode larvae were extracted using a Baermann funnel and counted under a microscope. Each fungus-nematode combination was replicated three times to calculate the efficiency of the fungi based on the number of nematodes captured and killed.

### 2.4 Statistical analysis

The results were statistically analyzed to determine significant differences between the nematophagous fungi species and nematode species. Mean and standard deviation were calculated using GraphPad Prism 10 (GraphPad Software, Inc., San Diego, USA). The data were then analyzed with a two-way ANOVA to examine the effects of fungal species and medium (agar plate vs. autoclaved soil) on the number of reduced larvae. Significance was set at an alpha level of 0.05. ANOVA was used to determine the main effects of the factors and their interaction. Where results were significant, a Tukey post-hoc test was performed to allow pairwise comparisons between groups.

## 3 Results

### 3.1 Efficacy of the fungi on agar plates

In a first experiment, the interaction between the three nematophagous fungi (*Arthrobotrys oligospora*, *Dactylaria scaphoides* and *Nematoctonus leiosporus*) and the three parasitic nematodes (*Strongyloides* sp., *Trichostrongylus* sp., and *Oesophagostomum* sp.) was investigated on agar plates.

#### 3.1.1 Mycelial growth and nematode interaction

*D. scaphoides* showed good mycelial growth on potato-carrot agar (half-strength). The growth of *A. oligospora* and *N. leiosporus* on potato-dextrose agar (PDA) was slower compared to the other fungi.

**Fig. 2:**
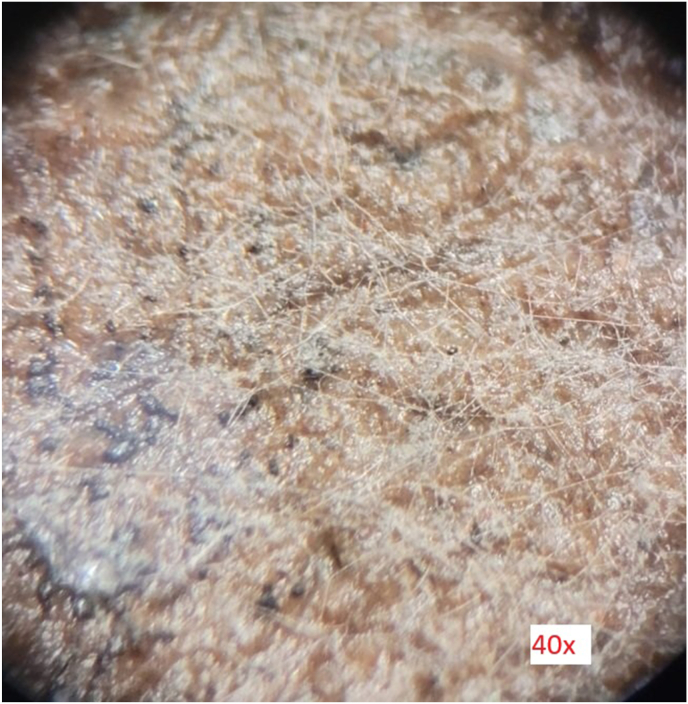
Growth of *Nematoctonus leiosporus* on the autoclaved enclosure soil. Binocular magnification approx. 40x.

After application of the parasites, an interaction was observed in all fungi in the first 48 h with regard to the capture of the parasitic nematodes (Fig. 2). The effectiveness of the individual fungi in trapping and killing nematodes varied during the 14-day observation period.

In the efficacy studies of different fungal treatments, *D. scaphoides* showed the highest efficiency against *Strongyloides* sp. by reducing the number of live nematodes L3 by an average of 75.33%. *A. oligospora* also proved effective but resulted in a slightly lower reduction of live nematodes L3 (54.67% on average). *N. leiosporus* was less effective compared to the other two treatments reducing the number of live nematodes L3 by an average of 25.33%. In the control group, which received no fungi, an average of 92% of nematodes L3 survived.

For *Trichostrongylus* sp., *D. scaphoides* was also the most effective treatment, with an average 70.0% reduction in nematode L3. *A. oligospora* and *N. leiosporus* also showed efficacy, but with lower average levels of nematode L3 killed (49.33% and 25.33% respectively). In addition, 8.0% of nematodes L3 died in the control group.

For *Oesophagostomum* sp., *D. scaphoides* reduced the number of live nematodes L3 by an average of 75.33% and was therefore the most effective treatment. *A. oligospora* also reduced the number of live nematodes L3 by 51.33%. *N. leiosporus* led to a lower reduction in nematode numbers, killing an average of 18.0% of nematodes L3. In this control group, an average of 95.33% of the nematodes L3 survived (Table 1).

**Table 1:** Efficacy of the fungi on agar plate and autoclaved enclosure soil after 14 days (each with three repetitions). The mean value [in %] after three repetitions are shown. Each time 50 larvae were applied. The number given is the percentage of reduced larvae observed in the corresponding nematophagous fungus.

Parasite	Fungi	Agar-Plate	Enclosure soil
		Mean [%]	Mean [%]
*Strongyloides* sp.	*Arthrobotrys oligospora*	54,67	62,00
	*Dactylaria scaphoides*	75,33	64,67
	*Nematoctonus leiosporus*	25,33	24,67
	Control	8,00	7,33
*Trichostrongylus* sp.	*Arthrobotrys oligospora*	49,33	54,67
	*Dactylaria scaphoides*	70,00	66,67
	*Nematoctonus leiosporus*	25,33	16,00
	Control	8,00	4,67
*Oesophagostomum* sp.	*Arthrobotrys oligospora*	51,33	58,00
	*Dactylaria scaphoides*	75,33	68,67
	*Nematoctonus leiosporus*	18,00	16,67
	Control	4,67	6,00

### 3.2 Effectiveness of fungi in autoclaved enclosure soil

In the second experiment, the interaction between the three nematophagous fungi (*A. oligospora, D. scaphoides* and *N. leiosporus*) and the three parasitic nematodes (*Strongyloides* sp.*, Trichostrongylus* sp. and *Oesophagostomum* sp.) was tested on autoclaved soil from wild animal enclosures in the laboratory to simulate more natural growth conditions.

#### 3.2.1 Mycelial growth and nematode interaction

**Fig. 3:**
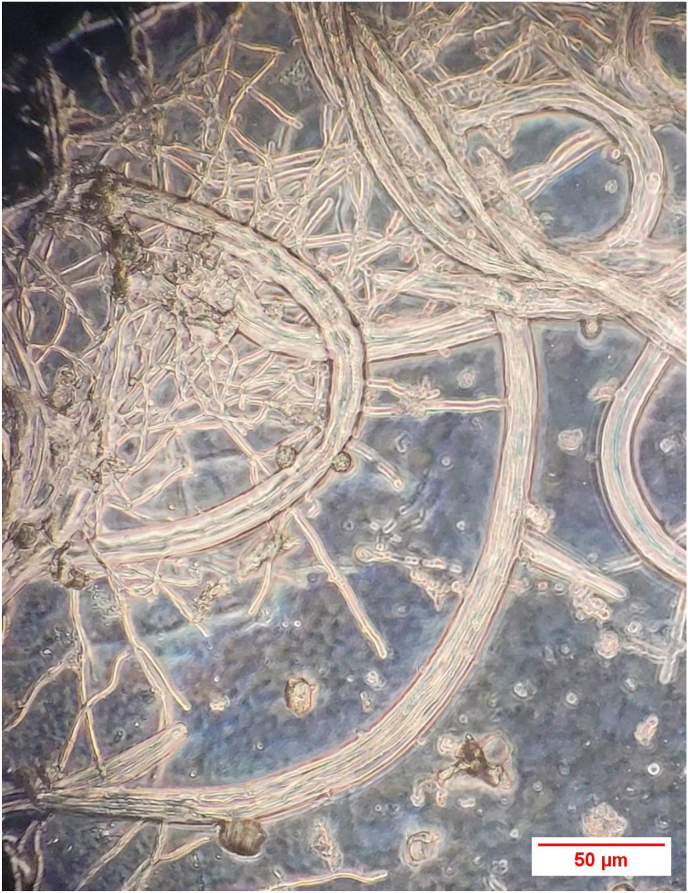
Interaction of *Nematoctonus leiosporus* with *Trichostrongylus* sp. *Nematoctonus leiosporus* attaches itself to the parasitic nematode by means of catch hyphae. Microscope magnification 60x.

*D. scaphoides* showed good mycelial growth on the enclosure soil. The nematophagous fungus quickly formed a fine network on the soil (Fig. 3). *A. oligospora* also exhibited good mycelial growth on the soil, albeit somewhat slower than *D. scaphoides.* The growth of *N. leiosporus* was significantly lower and remained rather localized.

After application of the parasites, a good interaction was observed with *D. scaphoides* and *A. oligospora* with regard to the capture of parasitic nematodes. The effectiveness of the individual fungi in catching and killing the nematodes varied similarly on the enclosure soil during the 14-day observation period.

In the trial with *Strongyloides* sp., *Dactylaria scaphoides* showed a relatively high efficacy, reducing the number of live nematodes L3 by an average of 64.67%.*A. oligospora* proved to be similarly effective, killing an average of 62% of nematodes L3. *Nematoctonus leiosporus* was less effective in this context and enabled the survival of an average of 75.33% of nematodes L3. In the control group, which received no fungi, the average survival of nematodes L3 was 92.67%, demonstrating the effectiveness of the fungal treatments.

In the tests with *Trichostrongylus* sp., *D. scaphoides* again proved to be the most effective treatment, with an average reduction in nematode L3 of 66.67%. *A. oligospora* also achieved a reduction in nematode L3 with an average of 54.67%, but was less effective than *A. oligospora*. *N. leiosporus* performed the worst, leaving an average of 84.0% live nematodes L3. In the control group, an average of 95.33% of nematodes L3 survived, emphasising the effectiveness of the fungal treatments compared to the untreated group.

In the trail with *Oesophagostomum* sp., *D. scaphoides* reduced the number of living nematodes L3 the most, namely by an average of 68.67%. *A. oligospora* was also effective, but with a slightly lower average of 58.0% of nematodes L3 killed. *N. leiosporus* again proved to be the least effective treatment with an average of 83.33% nematode L3 survival. In this control group, 94% of nematodes L3 survived.

Again, *D. scaphoides* was the most effective treatment (Table 1).

### 3.3 Comparative analysis

Two-factorial ANOVA revealed a significant interaction between fungal species and medium (F = 4.824, *P* = 0.0141), indicating that the effectiveness of the fungi in reducing larval numbers was dependent on the medium used. The main effect of fungal species was also significant (p < 0.0001), with *D. scaphoides* showing the highest overall efficiency by reducing the number of live larvae the most. The main effect of medium alone was not significant (F = 0.9767, *P* = 0.3377), suggesting that the average number of live larvae on agar plates and in autoclaved soil was comparable. The Tukey post-hoc test showed significant differences between the fungal species within each medium. On agar plates, *D. scaphoides* was significantly more effective than *A. oligospora* (p < 0.0001) and *N. leiosporus* (p < 0.0001). Compared to the control, all tested fungal species significantly reduced the number of larvae, with the largest reductions observed by *D. scaphoides*. There were also significant differences in the autoclaved soil: *D. scaphoides* showed a higher efficiency than *A. oligospora* (p = 0.0234), while *A. oligospora* significantly reduced the number of larvae compared to *N. leiosporus* (p < 0.0001). The comparison between media showed that the efficiency of *D. scaphoides* was higher on agar plates than in autoclaved soil (p = 0.0172), while *A. oligospora* was more effective on autoclaved soil (p = 0.0247). There were no significant differences between the media for *N. leiosporus* and the control. The comparative analysis of the effectiveness of the fungi on agar plates and autoclaved enclosure soil showed different patterns depending on the fungal species and nematode species*. D. scaphoides* was in most cases most effective on the agar plates, especially for the nematode species *Strongyloides* sp., *Trichostrongylus* sp. and *Oesophagostomum* sp. by reducing the number of surviving larvae the most. In contrast, *A. oligospora* proved to be consistently more effective on the autoclaved enclosure floor, while *N. leiosporus* showed the least effectiveness overall, regardless of the medium.

## 4 Discussion

The results of the study show different efficacies of the three nematophagous fungi tested on the three nematode species examined. The mean value of nematodes killed differs depending on the fungus and parasite, which indicates a specific effect of the fungi.

The experiments showed different patterns of effect between the agar plate and the enclosure floor. In the controlled environment of the agar plate, where ideal conditions such as better nutrient availability, controlled humidity and no competition from other microorganisms are present, the fungi were sometimes more efficient. In soil, a more complex and possibly less favourable environment, the efficacy of some fungi against certain parasitic larvae remained stable or was even higher. For example, *Arthrobotrys oligospora* was more effective against *Strongyloides* sp. and *Oesophagostomum* sp. in soil than on the agar plate. Despite these differences, the relative efficacy pattern of the fungi remained similar: *Dactylaria scaphoides* showed the highest efficacy in both environments. *Arthrobotrys oligospora* was more effective against some nematodes in soil than on the agar plate, while *Nematoctonus leiosporus* was the least effective in both media.

These results suggest that *D. scaphoides* has good potential as a biological control agent against parasitic nematodes. Koning et al. (1995) already observed a conspicuous predatory activity of *D. scaphoides* and *A. oligospora* as well as the formation of a three-dimensional adhesive network (Koning et al., 1996). Surprisingly, compared to *A. oligosphora*, and to the authors' knowledge, there are no studies investigating the use and efficacy of *D. scaphoides* as a biological control agent.

*Arthrobotrys oligospora* should be further investigated despite its lower effectiveness on the agar plate, as it was more effective on soil. This is because *A. oligospora* can be found in a variety of substrates, including animal feces, soil and compost. In addition, this and related species have been shown to be not only phytophagous, but also a biological control of gastrointenstinal parasites (Saumell et al., 2015; Wang et al., 2023). Several studies have reported the successful use of *Arthrobotrys* species fed to animals orally as pellets to control parasites (Szewc et al., 2021).

Unfortunately, *Nematoctonus leiosporus* was unable to achieve a noticeable effect either on the agar plate or on the enclosure soil. This may come as a surprise as this fungus is also widespread and can often be found in agricultural soils, feces and decaying plant litter. Furthermore, all *Nematoctonus* species possess predatory and parasitic mechanisms, unlike other nematophagous fungi genera. However, to the authors' knowledge, also no comparable studies have investigated the efficacy of *N. leiosporus* as a biological control agent. Therefore, it seems reasonable to further investigate the species and its mode of application (Koziak et al., 2007).

The control groups without fungal treatment showed low mortality, indicating that the media themselves did not cause significant nematode mortality. This confirms that the observed nematode mortality in the treated groups is largely due to the effect of the fungi.

Despite these interesting findings, the results must be interpreted with caution due to limitations. Potential limitations of this study include the use of a limited number of animal faecal samples and nematodes, and the number of replicates. Although the results represent the possible potential of the three nematophagous fungi, a larger sample size and additional replicates would be useful to further validate these results. There was also no counting of the number of chlamydospores. Instead, the amount of inoculum was determined using a standardised mycelium weight (approximately 5 g) per fungal species to provide a comparable baseline. However, this may have led to variations in the actual number of chlamydospores between samples, which could affect the reproducibility and comparability of the results. In addition, the tests were conducted under laboratory conditions, which may not reflect the complex interactions in the natural environment. Further field trials are needed to investigate both different conditions and times in relation to the efficacy of the fungi and the parasites of the captive animals. In addition, practical applicability in the field needs to be confirmed. Research into fungal species is of great importance. Studies report on a large number of fungi that occur naturally in the soil and have antagonistic methods for combating parasite eggs and larvae (Palomero et al., 2020). In addition, studies are constantly discovering new species that have the potential to act as biological controls against gastrointestinal nematodes (Saumell et al., 2015).

This is in stark contrast to the use of anthelmintics. Even if the frequent use of anthelmintics leads to an increase in resistance, they remain the most common method of combating parasitic infections for the time being. The reason for this is that the efficacy of products with nematophagous fungi is described as significantly lower than the efficacy of anthelmintics (Farah Haziqah et al., 2019; Li et al., 2022; Ojeda-Robertos et al., 2019; Szewc et al., 2021; Vieira et al., 2016). This is another reason why further field trials with the three fungi would be interesting.

In addition to the use of anthelmintics, quicklime is also used in some cases. By increasing the pH value and temperature of the soil, parasites in the soil can be killed (Capizzi-Banas et al., 2004). However, this method is expected to have a strong impact on the soil microbiome. Preventive measures are usually difficult to implement. Preventive hygiene measures, such as the regular collection of animal droppings, are often used (Hernández et al., 2018a). However, this can be complicated depending on the animal species and enclosure structure and size. The frequently used rotational grazing system is also difficult to implement in wildlife parks and zoos (Hernández et al., 2018a; Palomero et al., 2020).

The available data on the control of parasites with biological antagonists continues to grow and at the same time shows many possibilities, such as the use in soil, on feces and in animals with or without anthelmintics (Hernández et al., 2018b; Li et al., 2022; Luns et al., 2018; Palomero et al., 2020).

Several studies on the use of nematophagous fungi show that they can have valuable effects both on the soil microbiome and in terms of anthelmintic/anticoccidial reduction (Hernández et al., 2016, 2017; Lozano et al., 2023, 2024; Viña et al., 2022; Voinot et al., 2020, 2021). In his study, Hernández et al. (2016) shows that the use of feed pellets containing fungal spores can significantly reduce the excretion of worm eggs without affecting the health or immune system of the horses. The soil microbiome was also not negatively affected. Voinot et al. (2020) demonstrated a particular benefit. Their study in dairy cows investigated an integrative control strategy combining anthelmintics, feed pellets and rotational grazing. Daily ingestion of fungal spores, the effectiveness of the rotational grazing strategy and deworming demonstrated a sustained reduction in parasite egg production, which further reduced infection pressure on pastures and reduced reliance on chemical treatments. Hernández et al. (2017) also demonstrated the success of using different nematophagous fungi, such as *Mucor* spp. or *Trichoderma* sp., in fenced wildlife. The antagonistic effect against parasite eggs also made it possible to prevent their development in the soil, which reduced the spread of nematodes and promoted sustainable control in fenced wildlife areas. The results of the study by Lozano et al. (2024) are also groundbreaking. This study recently showed that the growth of *Mucor* spp. is unaffected by various anthelmintics and anticoccidials and could therefore have great potential in the control of avian infections, especially when used in combination with drugs and fungi. Therefore, nematophagous fungi are increasingly proving to be a useful and animal, human and environmentally friendly solution (Braga and De Araújo, 2014).

Soil-transmitted helminths will continue to pose a significant health threat to wildlife in enclosures, so further research into nematophagous fungi as an alternative solution to conventional anthelmintics is of great importance in order to develop sustainable and environmentally friendly control strategies.

## 5 Conclusion

In conclusion, our results show that the nematophagous fungus *Dactylaria scaphoides* has the greatest potential as a biological control agent against gastrointestinal parasites, although it has been little studied in this context. *Arthrobotrys oligospora* has also shown potential on soil and is well studied for its usefulness in other applications. *Nematophthora leiosporus* did not show sufficient efficacy in our trials, but to the authors' knowledge has also been little studied.

Nematophagous fungi are a promising alternative to conventional chemical control methods because of their unique mode of action, versatility and environmental friendliness. They could be particularly important for the control of parasitic nematodes. However, further research is needed to better understand and evaluate their efficacy under different environmental conditions and their cost-effectiveness.

## CRediT authorship contribution statement

**Christopher Sander:** Writing – original draft, Validation, Methodology, Investigation, Formal analysis, Data curation. **Stephan Neumann:** Writing – review & editing, Supervision, Resources, Funding acquisition, Conceptualization.

## Ethical standards

The authors assure that none of the procedures that contributed to this work involved animal testing, so the ethical standards are not applicable.

## Financial support

This project was funded by the Federal Ministry of Economy and Technology (Project ZIM ZF4351502MD9).

## Conflicts of interest

The authors declare no conflict of interest.
